# Human cardiac ^31^P magnetic resonance spectroscopy at 3T with a receive array: is single-loop or dual-loop RF transmission superior?

**DOI:** 10.1186/1532-429X-17-S1-P248

**Published:** 2015-02-03

**Authors:** Christopher T Rodgers, Mabel Li, Gillian MacNaught, Scott Semple

**Affiliations:** RDM Cardiovascular Medicine, University of Oxford, Oxford, UK; Department of Chemistry, University of Oxford, Oxford, UK; University of Edinburgh, Edinburgh, UK

## Background

Human cardiac phosphorous MR spectroscopy (^31^P-MRS) provides direct insight into cardiac energetics by measuring concentrations of ATP, ADP, phosphocreatine (PCr) and other species. Yet at 3T, excitation flip angles in the inferior segments of the myocardium have been limited to be much less than the "Ernst angle" needed to maximise spectral SNR. This has made it impossible so far to acquire spectroscopic images that cover the whole heart, which in turn has limited ^31^P-MRS to the study of diffuse rather than focal disease. In this study, we test whether splitting the RF transmission between anterior and posterior coils improves spectral quality across the myocardium compared to transmission from the anterior side alone.

## Methods

Data were acquired using a 3T Trio MRI scanner (Siemens) and two state-of-the-art 8-element cardiac receive array coils (Rapid Biomedical). Each comprised four anterior and four posterior 20x6 cm^2^ receive elements. One coil had only a 30x29 cm^2^ anterior transmit loop and the other had 30x29 cm^2^ transmit loops in both anterior and posterior pieces.

Normal volunteers (7 males, 23-41y, 65-82kg, 1.70-1.93m) were recruited with ethical approval. Cardiac ^31^P spectra were acquired with an ECG-triggered, 3D-UTE-CSI pulse sequence over a 35x35x35 cm^3^ FOV of 22x22x10 voxels, with 2 averages at k=0 and WSVD coil combination in a total of ~24min for a 70bpm subject. Using subject-specific B_1_ maps, the excitation voltage was set each time to give a 30° flip angle in the interventricular septum.

Spectra in each voxel were fitted with a custom Matlab implementation of AMARES, with prior knowledge specifying 11 Lorentzian peaks (α,β,γ-ATP multiplet components, PCr, PDE and 2x 2,3-DPG), with fixed amplitude ratios and scalar couplings for the multiplets. Spectral SNR was defined as peak height / baseline SD after application of a matched filter.

## Results

**Figure 1:**
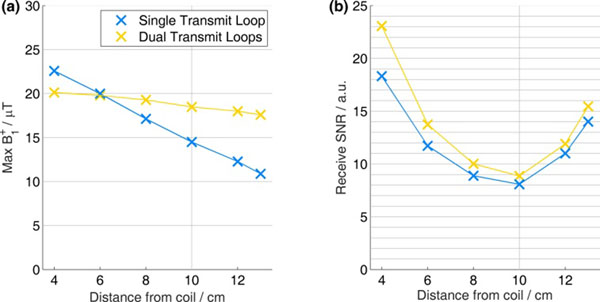
Comparison of coil transmit fields (B_1_^+^) and receive signal-to-noise ratios (SNR) using a purpose-built phantom comprising a 2x2x2cm^3^ cube of KH_2_PO_4(aq)_ in 14L of 73mM saline. The dual-transmit coil has a much more uniform B_1_^+^ than the single-transmit coil. The dual-transmit coil has greater B_1_^+^ than the single-transmit coil at depths >6cm. (The grey line marks the typical depth of the septum: ~10cm.) The receive SNR of the two coils is very similar, which is as expected given their matching design.

Across the phantom (Fig. 1), we observed B_1_^+^ variation of 50% for single-loop transmit, but only 10% with dual-loop transmit. The receive SNR at 10cm (the depth of the septum) differed by <10% between the coils (because they have identical receive circuitry).

**Figure 2:**
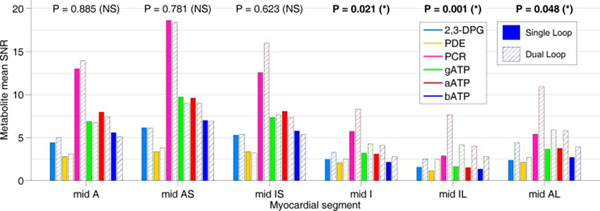
Comparison of mean ^31^P-MRS peak SNRs in-vivo in the mid-short axis segments for the two coils. Solid bars denote the single-transmit coil and cross-hatched bars denote the dual-transmit coil. P values from a paired t-test using all subject and all peaks are reported for each segment. In the regions of the heart closest to the surface (the anterior - inferoseptal segments) there is no significant difference between the performance of the two coils, but in the deeper regions of the heart (the inferior - anterolateral segments) the dual-transmit coil has significantly higher spectral SNR. The dual-transmit coil has a significant advantage for studies requiring whole-heart coverage.

In-vivo, both coils performed similarly in the anterior segments, but the dual-transmit coil had a significantly improved (P = 0.021, 0.001, 0.048) spectral SNR in the inferior cardiac segments relative to the single-loop coil. Even so, the SNR in the inferior cardiac segments obtained with this 24min protocol was still below that desirable for clinical studies with modest numbers of subjects at this point.

The first human cardiac 31P-MRS study at 7T saw a 2.8x SNR increase compared to 3T using the same coil geometry at both fields. Together with our findings here, this suggests that a dual-loop transmit coil at 7T will be able to achieve whole-heart coverage with clinically-acceptable SNR and total scan duration.

## Conclusions

Dual-loop transmit significantly improves the SNR in ^31^P-MRS of the inferior segments of the heart compared to single-loop transmit at 3T.

## Funding

Funded by a Sir Henry Dale Fellowship from the Wellcome Trust and the Royal Society [Grant Number 098436/Z/12/Z], the British Heart Foundation Centre of Research Excellence (CoRE) award (Edinburgh) and the Centre for Cardiovascular Sciences (Edinburgh).

